# Worldwide research productivity in emergency contraception: a bibliometric analysis

**DOI:** 10.1186/2054-7099-1-6

**Published:** 2015-05-05

**Authors:** Waleed M Sweileh, Sa’ed H Zyoud, Samah W Al-Jabi, Ansam F Sawalha

**Affiliations:** 1 Department of Pharmacology/Toxicology, College of Medicine and Health Sciences, An-Najah National University, Nablus, Palestine; 2 Department of Clinical and Community Pharmacy, College of Medicine and Health Sciences, Nablus, An-Najah National University, Nablus, Palestine

**Keywords:** Emergency contraception, Bibliometrics, Scopus

## Abstract

**Background:**

The main goal of this study was to assess worldwide research activity in emergency contraception (EC) using bibliometric indicators.

**Methods:**

Data in SciVerse Scopus were searched for documents pertaining to emergency contraception. Data obtained were then exported to Microsoft Excel and analyzed using Statistical Package for Social Sciences.

**Results:**

A total of 2142 documents were published about EC worldwide. Documents were written in 27 different languages and were published from 78 countries. Publications in EC started on late 1960s. Total number of citations for published EC documents was 30154 while median citation per document was six. The *h*-index of the retrieved documents was 58. The leading country in EC research was United States of America with a total of 559 documents (26.10%). One hundred and ninety five (9.10%) documents were published in *Contraception* journal. The leading institution in EC research and publications was Princeton University (50; 2.33%) followed by University of California, San Francisco (34; 1.59%).

**Conclusions:**

The present data revealed that there is a worldwide increasing interest in EC research. Willingness of health policy makers to make EC accessible to the public will determine the future of EC research activity and future of EC as a contraceptive method.

## Background

Contraception-related research has gained a lot of attention in the past few decades. The growth of population, rights of women to choose the time of motherhood, high prevalence of HIV disease, as well as the strong religious and political debate about abortion has created more attention and research momentum toward contraception science [1–4]. Furthermore, the economical and emotional burden of high rates of unintended pregnancies (UP) had drawn more attention and interest in novel and practical contraceptive methods. Historically speaking, women tried several behavioral and traditional contraceptive methods to avoid UP [5]. Currently there are several different natural, hormonal, mechanical and surgical methods for contraception that made a significant worldwide reduction in UP and illegal abortion [6–8]. However, despite the great advancement in contraception science and technology, no contraceptive method is 100% effective in preventing pregnancy all the time [9–11]. Furthermore, women might have sexual intercourse at unexpected times or they might be forced to have sex such as in rape situations. A practical contraceptive choice for women in such situations is the use of emergency contraceptive (EC) method. There are two main types of EC methods; emergency contraceptive pills (or called morning-after pill) and Intra Uterine Devices (IUD) that are sometimes used for the purpose of EC [12–15]. Emergency contraceptive pills are available as combined estrogen and progestin pills or progestin-only (levonorgestrel) pills, or antiprogestin (ulipristal acetate or mifepristone) pills [16–19]. In early 1970s, Dr. Albert Yuzpe was the first to prescribe birth control pills for emergency contraception [20]. In 1998, the FDA approved the marketing of the first EC product. One year later, the FDA approved the first progestin-only EC named Plan B® [21, 22].

Many review articles have been published about different types of contraceptive methods including emergency contraception [23–25]. However, scanning the literature showed no bibliometric studies about any type of contraceptive methods had been published. In contrast, hundreds of bibliometric studies in various medical fields and from different parts of the world had been published. Therefore, the goal of this bibliometric study was to assess the quantity and quality of EC–related research worldwide. Such a study will give insight into the current state of EC field. Furthermore, this study will provide baseline data for future similar projects in the field of contraception bibliometrics. So, in this study, we will give researchers in the in reproductive and family planning field an informative description of published literature about EC.

## Methods

### Search strategy

SciVerse Scopus, developed by Elsevier, was searched for data pertaining to EC. The choice of Scopus database was based on the fact that it is one of the largest available databases. In this study, title search strategy was applied. All subject areas in Scopus search engine including health sciences, social sciences, life sciences and physical sciences, were selected. The date range of the study was all years up to December 31st 2012. This time interval was selected to account for the time delay between the date an article is published and the date it appears within the Scopus database. Articles published after December 31st, 2012 were not consistently present within the database compared to earlier published articles., therefore articles after this date were excluded from statistical analysis. The key words used in the search query were those pertaining to emergency contraception. These key words were made after extensive review of published literature about emergency contraception. We used the following keywords: emergency contraception or postcoital contraception or morning-after-pill or emergency contraceptive pills or emergency hormonal contraception or postcoitus or post-coital contraceptive or Yuzpe or Plan B or Ulipristal acetate or levonorgestrel or intrauterine or IUD. These key words must be within articles that have the following phrase within Title/Abstract/keywords: “emergency contraception”. The key words or phrases used in search engine were related to emergency contraception. These key words were present in recent review articles about emergency contraception. Documents that were published as erratum were excluded. Scopus search engine has a function that gives a summary of the types of published documents. The authors did not do a manual search for erratum documents; rather, it was given by Scopus database itself.

### Indices of research productivity

Data were presented as rank order using the standard competition ranking (SCR). Only the ten top-ranked items were presented. The *h*-index, a marker of quality, was also presented. The *h*-index is the number of articles (h) that have received at least h citations [26]. Quality indicators considered for the top-ten ranked journals were the journal impact factor (IF) which was evaluated using the Journal Citation Report (JCR; Web of Knowledge) 2012 science edition by Thomson Reuters (New York, NY, USA) while the second indicator was the *SCImago Journal Rank* (SJR) indicator (Available at: http://www.scimagojr.com/SCImagoJournalRank.pdf).

### Ethical approval

The Institutional Review Board (IRB) at An-Najah National University considers that such study does not require an IRB application and approval since no human subjects are involved.

### Statistical analysis

Data from Scopus were exported to Microsoft Excel® and then transferred to the Statistical Package for Social Sciences, Version 15 (SPSS; SPSS Inc., Chicago, IL, USA) program for analysis. Categorical variables were expressed as Frequency and percentages. Categorical variables include document type, top 10 countries, institutions, authors and journals.

## Results

**Figure 1:**
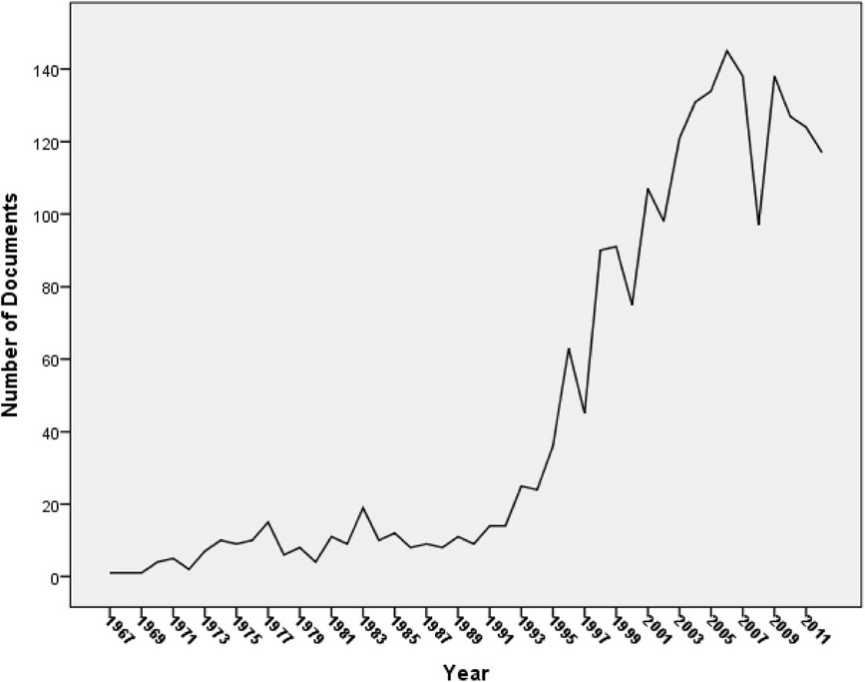
**Worldwide growth of EC research.**

**Figure 2:**
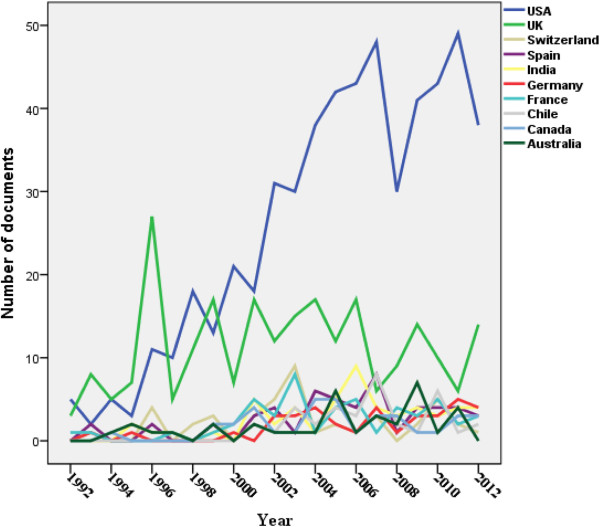
**Number of retrieved articles in emergency contraception with time for the top 10 countries.**

Using the methodology stated above, 2163 documents about EC were retrieved. Twenty one erratum documents were excluded. Therefore, analysis was confined to 2142 documents. Of the 2142 documents, there were 1202 (56.12%) original journal research articles, 291 (13.59%) review articles, 187 (8.73%) letters, 176 (8.22%) Note, 116 (5.42) short surveys, 74 (3.45%) editorials, 37 (1.73%) conference papers, 54 (2.52%) undefined documents, five (0.23%) book chapters (Table 1). Retrieved documents were written in 27 different languages and from 78 different countries. The main language of published documents was English (1830; 85.43%) followed distantly by Spanish (76; 3.54%) and French (64; 3.03%) languages. The annual number of documents published about emergency contraception remained low and steady until 1990 (Figure 1). Table 2 shows top 10 countries whose researchers published most about emergency contraception. One fourth of retrieved documents were published from the USA (559; 26.10%). India ranked third (62; 2.89%) in emergency contraception after USA and UK. In the past 20 years, the number of retrieved articles in EC showed an obvious increase by time in USA. However, for the other top 10 countries, the number of retrieved articles remained steady and did not show an obvious increase in the past 20 years (Figure 2). Seventy one documents (3.31%) were published by African countries while 15 (0.7%) documents were published from Middle Eastern Arab countries. The total number of citations for retrieved documents at the time of manuscript writing was 19671 with *h*-index of 58 and an average citation of 10.58 per document. In Table 3, a list of the top 10 cited documents about emergency contraception is shown [27–36]. Four of the top 10 cited articles were published in *Lancet* journal, 3 in *New England Journal of Medicine*, 1 in *Human Reproduction,* 1 in *British Medical Journal* and 1 in *Journal of the American Medical Association.* The highest number of citations recorded was 558 (Table 3). Table 4 shows top 10 journals in which documents about emergency contraception were published. A total of 195 (9.10%) retrieved documents were published in the journal *Contraception* whereas 58 (2.71%) were published in *Journal of Family Planning and Reproductive Health Care*. The leading institution in emergency contraception research and publication was Princeton University (50; 2.3%) followed by University of California, San Francisco (34; 1.59%); (Table 5). Most of the top 10 active institutions in the field of emergency contraception are based in USA and most are academic institutions. Of the 2142 documents, there were 75 documents having Yuzpe or Plan B in the title, 33 documents having ulipristal acetate in the title, 152 documents having levonorgestrel in the title, 65 documents having intrauterine device/IUD in the title, 118 documents having emergency contraceptive pills, and 34 documents having emergency contraception (Table 6).

**Table 1:** **Types of worldwide published document about EC**

Document type	Number	%
	N = 2142	
Article	1202	56.12
Review	291	13.59
Letter	187	8.73
Note	176	8.22
Short Survey	116	5.42
Editorial	74	3.45
Undefined	54	2.52
Conference Paper	37	1.73
Book Chapter	5	0.23
Total	**2142**	**100**

**Table 2:** **Top 10 countries in EC research**

SCR	Country	Number	%	World region
		N = 2142		
1st	United States of America	559	26.10	North America
2nd	United Kingdom	263	12.28	EU
3rd	India	62	2.89	Asia
4th	Spain	56	2.61	EU
5th	France	55	2.57	EU
6th	Switzerland	49	2.29	E
7th	Germany	43	2.01	EU
8th	Canada	43	2.01	North America
9th	Australia	39	1.82	Oceania
10th	Chile	37	1.73	Latin America

*Abbreviations*: *SCR* Standard Competition Ranking, *E* Europe, *UE* European Union.

**Table 3:** **Top 10 cited articles about EC as extracted from Scopus**

SCR	Author-Year	Title	Journal	Times cited
**1st**	Grimes et al. 1998	Randomised controlled trial of levonorgestrel versus the Yuzpe regimen of combined oral contraceptives for emergency contraception	*Lancet*	558
**2nd**	Von Hertzen, et al. 2002	Low dose mifepristone and two regimens of levonorgestrel for emergency contraception: A WHO multicentre randomised trial	*Lancet*	397
**3rd**	Glasier and Baird 1998	The effects of self-administering emergency contraception	*New England Journal of Medicine*	265
**4th**	Glasier et al. 1992	Mifepristone (RU 486) compared with high-dose estrogen and progestogen for emergency postcoital contraception	*New England Journal of Medicine*	213
**5th**	Piaggio et al. 1999	Timing of emergency contraception with levonorgestrel or the Yuzpe regimen	*Lancet*	210
**6th**	Raine et al. 2005	Direct access to emergency contraception through pharmacies and effect on unintended pregnancy and STIs: A randomized controlled trial	*Journal of the American Medical Association*	203
**7th**	Glasier 1997	Emergency postcoital contraception	*New England Journal of Medicine*	201
**8th**	Ho and Kwan 1993	A prospective randomized comparison of levonorgestrel with the Yuzpe regimen in post-coital contraception	*Human Reproduction*	200
**9th**	Von Hertzen, and Van Look 1999	Comparison of three single doses of mifepristone as emergency contraception: A randomised trial	*Lancet*	171
**10th**	Webb et al. 1992	Comparison of Yuzpe regimen, danazol, and mifepristone (RU486) in oral postcoital contraception	*British Medical Journal*	170

**Table 4:** **Top 10 journals for EC publications**

SCR	Journal	Number	%	IF*	SJR
		N = 2142			
1st	*Contraception*	195	9.10	3.090	1.47
2nd	*Journal of Family Planning and Reproductive Health Care*	58	2.71	1.154	0.33
3rd	*Obstetrics and Gynecology*	54	2.52	4.798	1.9
4th	*European Journal of Contraception and Reproductive Health Care*	41	1.91	1.808	0.58
5th	*British Journal of Family Planning*	34	1.59	NA	NA
5th	*Lancet*	34	1.59	39.060	7.07
5th	*British Medical Journal*	34	1.59	17.215	1.48
8th	*Journal of Pediatric and Adolescent Gynecology*	26	1.21	1.630	0.41
8th	*Perspectives on Sexual and Reproductive Health*	26	1.21	1.708	0.87
9th	*Human Reproduction*	25	1.17	4.670	2.17

*Abbreviations*: *SCR* Standard Competition Ranking, *NA* not available, *IF* impact factor, *SJR* Standard journal ranking, *NA* not available, the journal was discontinued by year 2000.

*The impact factor was reported according to Institute for Scientific Information (ISI) journal citation reports (JCR) 2012.

**Table 5:** **Top 10 highly productive institutions in EC research**

SCR	Institution	Number	%	Country
		N = 2142		
1st	Princeton University	50	2.33	USA
2nd	University of California, San Francisco	34	1.59	USA
2nd	Family Health International	34	1.59	USA
4th	Organisation Mondiale de la Sante	32	1.49	Switzerland
5th	University of Utah	22	1.03	USA
6th	Population Council Headquarters	21	0.98	USA
6th	Population Council, Mexico City	21	0.98	Mixico
6th	Instituto Chileno de Medicina Reproductiva	21	0.98	Chile
9th	University of Edinburgh	20	0.93	UK
10th	All India Institute of Medical Sciences	17	0.79	India
10th	Karolinska University Hospital	17	0.79	Sweden

*Abbreviations*: *SCR* Standard Competition Ranking, *USA* United States of America, *UK* United Kingdom.

**Table 6:** **Common words used as a title in the retrieved documents about emergency contraception**

No.	Word or phrase	Frequency (%)*
1	“emergency contraception”	1436 (67)
2	“morning after”	401 (18)
3	‘levonorgestrel”	147 (6)
4	“emergency contraceptive pills”	118 (5)
5	“Intrauterine device” or “IUD”	65 (3)
6	“Yuzpe” or “Plan B”	75 (3.5)
7	“Post-coital”	52 (2)
8	“emergency hormonal contraception”	34 (1.6)
9	“Ulipristal acetate”	33 (1.5)

*total % exceeds 100 because there are some overlaps in results.

## Discussion

Appropriate use of contraceptive methods requires an understanding of how scientific research about efficacy and safety of various contraceptive methods has progressed. One method to assess past and current status of a particular medicine or procedure is to assess research output. As planned motherhood is now a pre-requisite for successful pregnancy and maternal health, emergency contraception has become more of a necessity for women [37–39]. Our study investigated research productivity about emergency contraception up until the year 2012 using Scopus as a search engine and as a source database. The total number of emergency contraception documents retrieved and analyzed using the methodology stated was 2142. This number represents a close approximation of worldwide research productivity in the field but definitely does not represent 100% of global publications about emergency contraception. Scopus is considered a trustful and powerful search engine with citation analysis. However, not all journals are indexed in Scopus and therefore publications pertaining to emergency contraception in these un-indexed journals were not counted. Nevertheless, this study and up to the author’s best knowledge is the first to do a bibliometric analysis of worldwide contraception-related research. The finding that the h – indexed of published emergency contraception documents is 58 indicates that there is a large audience of these published documents and citation in the regard is relatively high. There is a debate about the use of h-index as a marker of research quality. The h-index is commonly used instead of other simple bibliometric indicators like total number of papers or total number of citations. The h-index simultaneously measures the quality and quantity of scientific output. However, *h*-index measured using different databases can give different values and therefore each database has pros and cons when measuring the h-index [40–42]. Our study showed that several non-academic institutions, particularly non-profit organization, ranked among top 10 in emergency contraception research indicating that emergency contraception is a an issue of increasing interest from a demographic and family planning point of view. In addition, debate about emergency contraception and its potential abortion effect gave further momentum to research in this field. Some academic institutions have run an online website for emergency contraception awareness and directions [43]. Other institutions, like Population Council in Mexico, have dedicated their efforts to improve reproductive health through high-quality research in family planning, pregnancy-related problems including women’s rights to safe and legal abortion [43]. Of the top cited documents in emergency contraception, it was clear that research groups in Edinbrugh, UK and Geneva, Switzerland have produced highly citable documents in EC field.

Our study further showed that although the prevalence of UP, HIV and sexual assaults that included rape incidences were high in Africa during civil wars, the EC research in Africa remained low [44–48]. South Africa has some research contribution in EC research that addresses awareness and services pertaining to emergency contraception [49–53]. In contrast to other world regions, EC research is limited in Arab Muslim countries in the Middle East. A study indicated that awareness of women in Arab world regarding emergency contraception methods was very low [54]. Another study indicated that in contrast to English EC website users, the users of Arabic EC web sites were interested in the different aspects of EC than English website users [55]. The authors of the study suggested that there is a need for creating culturally specific EC website content for health education [55].

The future of emergency contraception seems promising given the upward increase in research output in the field. In the past 2 decades, the number of documents in EC has increased 20 folds. The popularity and future market and clinical use of EC seems to depend on various factors including religious and political debate in addition to safety and efficacy reports. Several studies have suggested that making EC methods available will positively affect the population growth and will make the future of such method more convincing [56, 57]. Definitely, the arguments concerning the mechanism of action of emergency contraception, particularly those pertaining to mifepristone, ulipristal acetate and levonorgestrel which act by inhibiting ovulation rather than implantation will overcome many religious barriers for use of EC [58]. The time course and clinical experience with emergency contraception in the past 30 years or so have shown a good safety profile of emergency contraception given to women a second chance to terminate an unwanted pregnancy [59]. Our study has few limitations that need to be listed. First of all, we used Scopus database and therefore documents published in non-Scopus-indexed journals were not included. Another limitation is that some articles did not contain the selected key words has been excluded. Therefore, the number of publications analysed in this study might not represent 100% of EC-based worldwide research activity. It should also be noted that the research output for certain institutes could have been under-estimated due to different English spelling in different articles. Therefore, such institutes might have two or more institute’s profiles in Scopus because their names were written differently in different documents. Finally, some documents might be counted twice especially those presented as conference material and then published as original research articles.

## Conclusion

The present data reveal a good bulk and rising trend of research activity about emergency contraception. As expected, the quantity of EC research activity was skewed toward USA and European countries. However, centres in Latin America and India have made good contribution to research in EC. Research activity and use of EC can be improved by increasing awareness and acceptability of such method among women and health policy makers.

## Abbreviations

SPSS: Statistical Package for Social Sciences
ISI: Institute for Scientific Information
JCR: Journal Citation Report
IFs: impact factors
IRB: Institutional Review Board
SCR: Standard Competition Ranking
USA: United States of America
UP: Unintended pregnancies
EC: Emergency contraceptive
IUD: Intra Uterine Devices.
